# Radiosensitization with an inhibitor of poly(ADP-ribose) glycohydrolase: A comparison with the PARP1/2/3 inhibitor olaparib

**DOI:** 10.1016/j.dnarep.2017.11.004

**Published:** 2018-01

**Authors:** Polly Gravells, James Neale, Emma Grant, Amit Nathubhai, Kate M. Smith, Dominic I. James, Helen E. Bryant

**Affiliations:** a Academic Unit of Molecular Oncology, Sheffield Institute for Nucleic Acids (SInFoNiA), Department of Oncology and Metabolism, University of Sheffield, Beech Hill Road, Sheffield, S10 2RX, United Kingdom; b Drug and Target Discovery, Department of Pharmacy and Pharmacology, University of Bath, Claverton Down, Bath, Somerset, BA2 7AY, United Kingdom; c Drug Discovery Unit, Cancer Research UK Manchester Institute, The University of Manchester, Wilmslow Road, Manchester, M20 4BX, United Kingdom

**Keywords:** PARP, PARG, Tankyrase, Radiosensitization, Homologous recombination, Non-homologous end-joining

## Abstract

Upon DNA binding the poly(ADP-ribose) polymerase family of enzymes (PARPs) add multiple ADP-ribose subunits to themselves and other acceptor proteins. Inhibitors of PARPs have become an exciting and real prospect for monotherapy and as sensitizers to ionising radiation (IR). The action of PARPs are reversed by poly(ADP-ribose) glycohydrolase (PARG). Until recently studies of PARG have been limited by the lack of an inhibitor. Here, a first in class, specific, and cell permeable PARG inhibitor, PDD00017273, is shown to radiosensitize. Further, PDD00017273 is compared with the PARP1/2/3 inhibitor olaparib. Both olaparib and PDD00017273 altered the repair of IR-induced DNA damage, resulting in delayed resolution of RAD51 foci compared with control cells. However, only PARG inhibition induced a rapid increase in IR-induced activation of PRKDC (DNA-PK) and perturbed mitotic progression. This suggests that PARG has additional functions in the cell compared with inhibition of PARP1/2/3, likely via reversal of tankyrase activity and/or that inhibiting the removal of poly(ADP-ribose) (PAR) has a different consequence to inhibiting PAR addition. Overall, our data are consistent with previous genetic findings, reveal new insights into the function of PAR metabolism following IR and demonstrate for the first time the therapeutic potential of PARG inhibitors as radiosensitizing agents.

## Highlights

• PARG and PARP inhibition both radiosensitize.
• PARP and PARG inhibition both alter the DNA damage response following irradiation (IR).
• PARP and PARG inhibition both alter homologous recombination following IR.
• Only PARG inhibition induces rapid activation of non-homologous end-joining post-IR.
• Only inhibition of PARG causes accumulation of cells in metaphase post-IR.

## Graphical abstract

**Figure:**
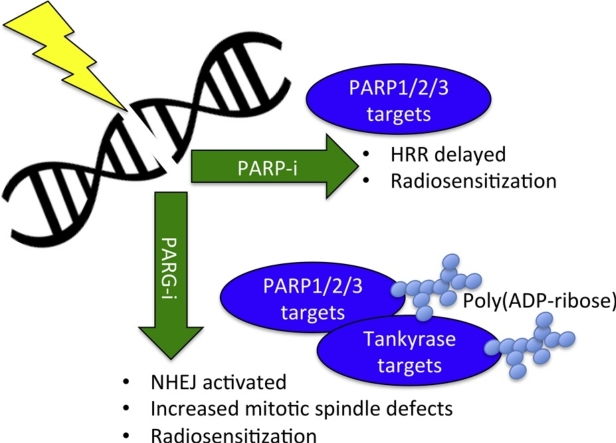

## 1 Introduction

The poly(ADP-ribose) polymerase (PARP) family of enzymes are recruited to, and activated at, sites of DNA damage, where they add poly(ADP-ribose) (PAR) to themselves and to other DNA repair and chromatin-remodeling factors [1], [2]. Once synthesised the PAR polymer is thought to act as a signal to recruit repair factors to the damage. In this way PARP proteins are considered to play a key role in coordinating the repair of single [3], [4], [5], [6], [7], [8], [9], [10] and double strand DNA breaks [11], [12], [13], [14], [15], and in the restart of stalled or collapsed DNA replication forks [16], [17], [18]. Given this key function in DNA repair, several inhibitors of the PARP proteins are now under development for cancer treatment, to be used either alone [19] or in combination with DNA damaging agents such as radiotherapy (reviewed in [20]). PARP1 depletion has been shown to modestly increase sensitivity to ionising radiation (IR) in mouse models [21], [22]. In addition, a variety of PARP inhibitors, reportedly targeting PARPs 1, 2 and 3 to various degrees, have been demonstrated to radiosensitize a variety of human tumour cell lines [23], [24], [25], [26], [27] including breast cancer [28], [29], [30], [31], and have shown success in several preclinical and clinical trials [32], [33], [34], [35], [36], [37], [38], [39], [40], [41], [42], [43]. Radiosensitization by these inhibitors is generally considered to be a replication dependent event [44], [45].

The catalytic action of all PARPs are reversed by the endo- and exoglycosidase activities of poly(ADP-ribose) glycohydrolase (PARG) [46], [47], [48], [49], [50], and it is proposed that following recruitment of other repair proteins to sites of damaged DNA, PAR must be removed for DNA repair to be completed [6]. Consistent with a role in DNA repair, PARG deficient cells have been reported to display reduced efficiency of double strand break (DSB) [51], [52], [53] and single strand break (SSB) repair [6], and to have difficulties during situations of replication stress [53], [54], [55], [56]. These defects in repair/replication suggest that PARG like PARP is a possible target as a single agent in certain genetic backgrounds [53] and for sensitizing to DNA damaging agents. The reported chemosensitizing effects are variable [6], [52], [57], [58], [59], [60], [61], but gene depletion or silencing of PARG using siRNA has consistently resulted in sensitivity to ionising radiation (IR) in mouse ES cells [62], [63] and human cancer cell lines [51], [64], with accumulation of mitotic defects and death occurring by mitotic catastrophe [51], [64].

Each of the radiosensitizing studies above was carried out in cells deficient in PARG, and while supportive, the investigation of the therapeutic potential of PARG has been limited by the lack of a cell permeable, specific, PARG inhibitor. Recently, we developed a novel, first in class, PARG inhibitor – PDD00017273 [65], which showed synthetic lethal killing in cells deficient in certain homologous recombination associated proteins [66]. Here we test the ability of the same agent to sensitize breast cancer cells to IR. In addition, we compare this with the radiosensitizing effects of olaparib. Olaparib has reported IC_50_ values of 5 nM, 1 nM and 4 nM for PARP1, PARP2 and PARP3 respectively [67].

## 2 Materials and methods

### 2.1 Cell culture

The MCF-7 and MDA-MB-231 breast epithelial adenocarcinoma cell lines were purchased from the American Type Culture Collection (ATCC^®^ HTB-22™ and ATCC^®^ HTB-26™ respectively). Cell lines were maintained in Dulbecco’s modified Eagle Medium (DMEM, Gibco, ThermoFisher Scientific, MA, USA) supplemented with 1× non-essential amino acids (NEAA, Sigma-Aldrich, MO, USA) and 10% Foetal bovine serum (Gibco) at 37 **°**C under an atmosphere containing 5% CO_2_.

### 2.2 Inhibitors

The PARG inhibitor, PDD00017273, [65] was resuspended in dimethyl sulfoxide (DMSO) at a concentration of 20 μM and stored at −20 **°**C. A final concentration of 0.3 μM was used. The PARP inhibitor, olaparib, was purchased from Cambridge Biosciences (UK) and prepared in DMSO to give a 1000× stock. A final concentration of 1 μM was used. The dual-site binding tankyrase inhibitor – 8-Methyl-2-(3-oxo-3-(4-((quinolin-8-yl)aminocarbonyl)-phenylamino)propyl)quinazolin-4-one (compound 14 in reference [68]) was prepared as a 5 mM stock in DMSO. A final concentration of 5 μM was used.

### 2.3 SiRNA transfection

ON-TARGETplus siRNA was purchased from Dharmacon (GE Healthcare Life Sciences, CO, USA) for two individual PARG (NM_003631) siRNA oligonucleotides, PARP1 (NM_001618) and the non-targeting siRNA #1 (scramble) control. All siRNAs were resuspended at 20 μM in 1 × siRNA universal buffer (Dharmacon) and stored at −20 **°**C. Cells were seeded in 6-well plates and left overnight to attach. The following day, cells were transfected with 20 nM siRNA (final concentration) using Dharmafect 4 reagent (Dharmacon) following manufacturers’ instructions. Knockdown was confirmed after 48 h by western blotting.

### 2.4 Clonogenic survival assay

Cells were plated at known densities in 90 mm dishes and left to attach for 4 h. After this time, inhibitors were added to the media at the concentrations stated above. The next day, cells were exposed to increasing doses of IR using an IBL437C Irradiator (Source 51.5TBq, Cs137) and then left for 15 days to form colonies. Colonies were stained with 4% methylene blue in 70% methanol and counted. Where siRNA knockdown was used, cells were transfected in 6-well plates and left for 48 h before replating at known densities in 90 mm dishes and exposing to increasing doses of IR.

### 2.5 Western blotting

Cells were lysed in RIPA buffer (50 mM Tris-HCl, 150 mM NaCl, 1% Triton X-100, 0.1% SDS, 1 mM EDTA, and 1% sodium deoxycholate) in the presence of 1× protease and phosphatase inhibitor cocktails (Roche, Sigma-Aldrich, MO, USA). An aliquot of 30 μg total protein (measured by BioRad DC protein assay) was run on an SDS-PAGE gel and transferred to Hybond ECL membrane (GE Healthcare, CO, USA). This membrane was immunoblotted with antibodies against Poly(ADP-ribose) 10H (1:400, Enzo Life Sciences, NY, USA), PARG (1:500, Santa Cruz Biotechnology, TX, USA), PARP1 (1:1000, Santa Cruz Biotechnology) and TUBB (β-tubulin; 1:2000, Sigma-Aldrich), each diluted in 5% milk and incubated at 4 **°**C overnight. After the addition of the appropriate HRP-conjugated secondary antibody and further washes, the immunoreactive protein was visualised on Hyperfilm™ ECL (GE Healthcare) using ECL reagents (GE Healthcare) following manufacturer’s instructions.

### 2.6 Immunofluorescence

Cells were plated on to coverslips and allowed to settle before treating with inhibitors overnight. The next day, cells were irradiated at 3 Gy and then either fixed immediately or left to repair at 37 **°**C for the time stated in the figures. Cells were fixed in 4% paraformaldehyde solution (Insight Biotechnology Ltd, UK) for 20 min at room temperature and then extensively washed (3 × 5 min in tris-buffered saline (TBS), 1 × 10 min in phosphate-buffered saline (PBS) containing 0.5% Triton X-100 and 3 × 5 min in TBS). Coverslips were placed in 10% goat serum (ThermoFisher Scientific) in TBS for 1 h at room temperature to block followed by a further 3 × 5 min washes in TBS prior to incubation with the primary antibodies anti-γH2AX (ser139) (Cell Signaling, MA, USA), RAD51 (Santa Cruz Biotechnology), DNA-PKcs pS2056 (Abcam, UK) or poly(ADP-ribose) 10H (Enzo Life Sciences), Pericentrin (Abcam), β-Tubulin (Sigma-Aldrich) or the PAR binding reagent MABE1016 (Millipore) each diluted (1:500) in TBS containing 3% goat serum, for 16 h at 4 **°**C. The coverslips were subsequently washed 4 × 10 min in TBS followed by incubation with the secondary antibodies, Alexa-fluor 594 goat anti-rabbit IgG (ThermoFisher Scientific) or Alexa-fluor 488 goat anti-Mouse IgG (ThermoFisher Scientific) diluted in TBS containing 3% goat serum (1:500) for 1 h at room temperature and finally washed 3 × 5 min TBS. Coverslips were then mounted onto microscope slides with DAPI containing mountant (Vector Labs, CA, USA).

All images were obtained with a Zeiss LSM 510 inverted confocal microscope using planapochromat 63 × /NA 1.4 oil immersion objective and excitation wavelengths 488 nm, 546 nm and 630 nm. Through focus maximum projection, images were acquired from optical sections 0.5 μM apart and with a section thickness of 1.0 μm. Images were processed using Adobe Photoshop (Abacus Inc.). The frequency of cells containing foci was determined by counting at least 100 nuclei on each slide.

### 2.7 Identification of mitotic phenotypes

Cells were stained with antibodies against TUBB (β-Tubulin), PCNT (pericentrin) and DAPI and observed by fluorescence microscopy. Mitotic cells were classified into prophase, metaphase, anaphase and telophase stages based on DAPI staining of the DNA (example images of classification are shown in Supplementary Fig. 1). β-tubulin stained spindle formation was classed as abnormal if it was either monopolar, asymmetric or disorganized. Pericentrin was used to allow identification of multipolar, monopolar or fragmented centrosomes.

### 2.8 Micronuclei scoring

Micronuclei were identified by DAPI staining in cells stained for γH2AX in the samples treated with inhibitors alone and 12 h post-IR exposure. Cells with greater than five micronuclei where regarded as necrotic and therefore not included in the analysis. Micronuclei were then scored as either negative or positive for γH2AX staining and average number of micronuclei of either type was calculated from the total number of cells counted.

### 2.9 pH3 staining and cell cycle analysis

Cells were seeded in 90 mm dishes and left to attach for 4 h before inhibitors were added to the media. The next day cells were exposed to 3 Gy IR and then left for 24 h before fixing in 70% methanol and stored overnight at −20 **°**C. After washing in PBS cells were resuspended in 2 ml PBS supplemented with 0.5% BSA (Sigma-Aldrich) and 0.25% Triton-X100. Following 15 min incubation on ice, cells were resuspended with Histone H3 pS10 antibody (Abcam, 1:1000) diluted in 100 μl of PBS supplemented with 0.5% BSA and 0.25% Triton-X100 and incubated for 2 h. After this time cells were washed with 0.25% Triton-X100 in PBS and then incubated with secondary antibody Alexfluor 488 goat anti-mouse IgG (1:100, diluted in 100 μl PBS supplemented with 1% BSA) for 30 min protected from light. Following a final wash with PBS, cells were incubated with 5 μl RNaseA (2 mg/ml) and 200 μl propidium iodide (PI, 50 μg/ml) for 15 min in the dark. Samples were analysed by flow cytometry using the FACSCalibur 488 nm laser (BD Biosciences, CA, USA).

### 2.10 Statistical analysis

Where p values are indicated the Student’s *T*-test was used for analysis between two sets of data, in each case two-sided, unpaired tests were carried out.

## 3 Results

### 3.1 Both PARP1/2/3 and PARG inhibitors increase sensitivity to ionising radiation

**Fig. 1:**
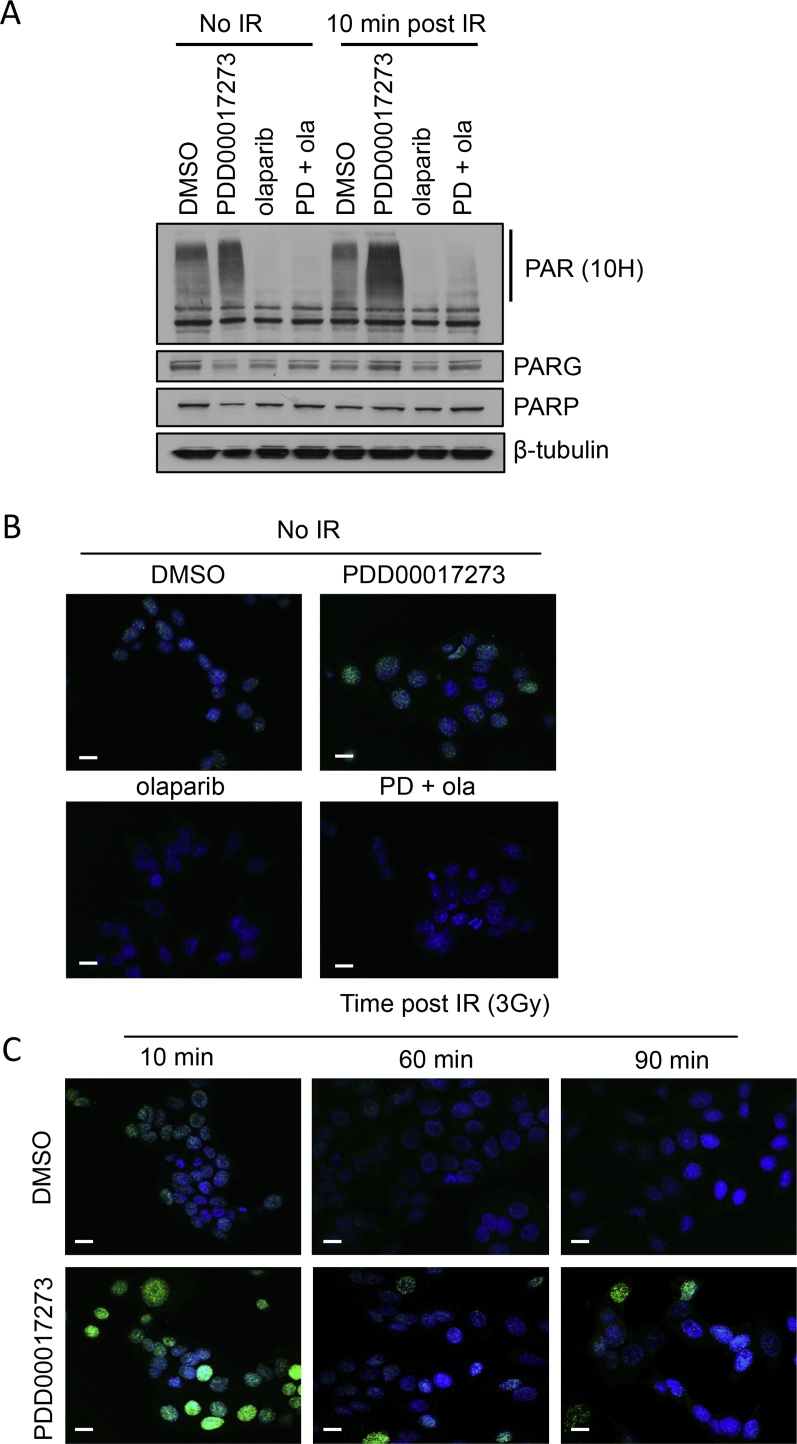
Inhibition of PARG leads to accumulation and persistence of poly(ADP-ribose) both alone and following ionising radiation. **(A**) Protein expression of poly(ADP-ribose (PAR), PARG and PARP, following inhibition of PARG with 0.3 μM PDD00017273, PARP with 1 μM olaparib, or both, either alone or post 3 Gy ionising radiation (IR). (**B&C**) Immunodetection of PAR in cells treated with inhibitor and left for various times post-IR as indicated. In all cases MCF-7 cells were incubated for 16 h with inhibitor before exposure to IR.

We previously established that 0.3 μM of the PARG inhibitor PDD00017273 is the optimum dose for inhibition of endogenous PARG activity, with minimal cell killing in the breast cancer cell line MCF-7 [53]. Here, the ability of PDD00017273 to inhibit PARG and lead to accumulation of PAR was confirmed by western blotting and immunofluorescent staining (Fig. 1A and B). In contrast, and as expected, incubation with olaparib led to reduced levels of endogenous PAR. Concomitant exposure to olaparib and PDD00017273 also resulted in reduced PAR accumulation, confirming the specificity of the PARG inhibitor and the reagents.

For therapeutic relevance a relatively low dose of 3 Gy IR was chosen. Ten minutes post-IR increased PAR could be detected using immunofluorescence but not by western blotting (Fig. 1A and C). However, in the presence of the PARG inhibitor a large increase in PAR was observed using both techniques, suggesting that PAR synthesis is induced by IR, but that the turnover is too rapid to always observe in control cells. IR induced PAR activity had returned to basal levels 10 min post-IR in DMSO treated cells, and remained high in a subset of cells at 90 mins post-IR when PARG was inhibited (Fig. 1C). Addition of olaparib or olaparib plus PDD00017273 during IR treatment led to reduced PAR (Fig. 1A). These data demonstrate that inhibition of PARG with PDD00017273 results in reduced PAR turnover and hence DNA damage-induced accumulation and persistence of PAR.

**Fig. 2:**
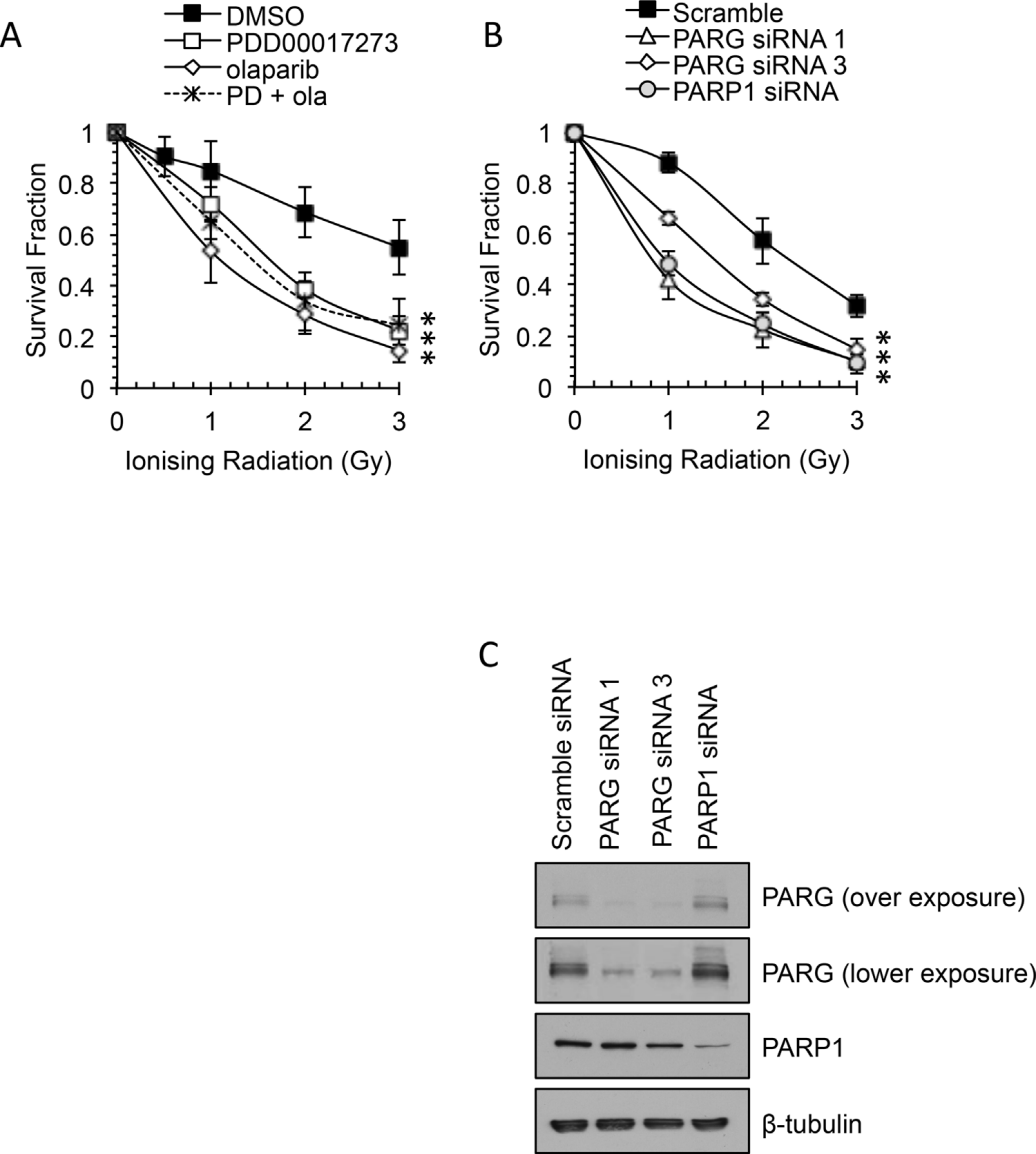
Inhibition or depletion of PARP or PARG increases sensitivity to ionising radiation. **(A**) Survival fraction of MCF-7 cells untreated (DMSO), treated with PARG inhibitor (PDD00017273), PARP inhibitor (olaparib), or both for 16 h prior to and during recovery from ionising radiation (IR). (**B**) Survival fraction following IR of siRNA transfected cells as indicated. Survival was measured by clonogenic survival assay. Mean and standard deviation of three independent repeats are shown. Statistical significance calculated by Student’s *T*-test, cf. to DMSO or scrambled siRNA control, where *** represents p < 0.001. (**C**) Protein expression of siRNA transfected cells 48 h post-transfection.

Previous genetic studies demonstrated that PARG has potential as a radiosensitizing target, but to date a therapeutic agent has not been available to test this hypothesis. Having demonstrated that PARG inhibition by PDD00017273 effects PAR turnover following IR, the PARG inhibitor was added to MCF-7 cells 16 h prior to treatment with IR and survival determined by clonogenic survival assay (Fig. 2A). PARG inhibition resulted in approximately 2–3 fold increase in sensitivity to IR compared with DMSO control (3 Gy – 22% survival vs. 55% in control; p < 0.0001). Consistent with this, depletion of PARG also caused reduced survival in response to IR (Fig. 2B). The effect induced was similar to that seen with olaparib or following co-treatment with both inhibitors (3 Gy – 15% and 25% survival respectively; p < 0.0001 compared with DMSO control). Likewise comparing PARP1 depleted cells with PARG depleted cells, there was no consistent difference in the degree of radiosensitization induced (Fig. 2B). Western blotting confirmed siRNA-mediated depletion of PARP1 and PARG (Fig. 2C). A similar effect was seen in the triple negative breast cancer (TNBC) cell line MD-MBA-231 (Supplementary Fig. 2). Our data support the idea that inhibition of PARPs can sensitize to IR in breast cancer, including both ER+ and TNBC [28], [29], [30], [31]. In addition, we show for the first time that a specific PARG inhibitor, PDD00017273, can sensitize to IR to a similar degree in these backgrounds.

### 3.2 PARP1/2/3 inhibition slows and PARG inhibition speeds up repair of IR induced DNA double strand breaks

**Fig. 3:**
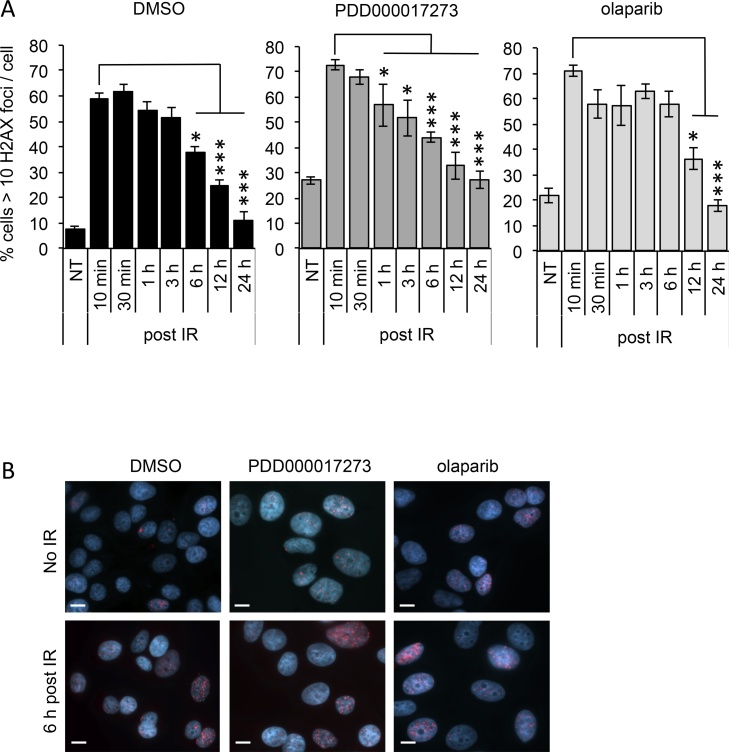
PARG and PARP inhibitors have different effects on repair of ionising radiation-induced DNA damage. (**A**) Percentage of cells displaying >10 γH2AX foci/cell in untreated (DMSO), PARG inhibited (0.3 μM PDD00017273), or PARP inhibited (1 μM olaparib) MCF-7 cells in the absence and at various times post 3 Gy ionising radiation (IR). Mean and standard error of the mean of three independent repeats is shown. Statistical significance calculated by two-sided Student’s *T*-test, cf. peak H2AX activation under same conditions, where *, ** and *** represent p < 0.05, 0.01 and <0.001. (**B**) Example images of γH2AX foci (Red) co-stained with DAPI. MCF-7 cells were incubated for 16 h with inhibitor before exposure to IR.

Ionising radiation induces breaks in DNA [69]. Given the proposed function of PARG in promoting efficient DNA repair, we examined induction and repair of DNA damage. To do this we used phosphorylation of histone H2AX on Ser139 (γH2AX) as a marker of DNA damage, and followed the kinetics of repair with time post-IR in the presence or absence of PDD00017273 or olaparib (Fig. 3). Consistent with our published data [53], [66], [70] inhibition of PARP1/2/3 or PARG resulted in a significantly increased basal level of γH2AX foci staining (p < 0.05 for PARP and p < 0.001 for PARG inhibitors compared with control). As expected, treatment with 3 Gy IR induced a rapid increase in the percentage of cells displaying greater than 10 γH2AX foci/cell (p < 0.001 at 10 min for each condition compared with corresponding mock irradiated sample). Considering the increased background staining, the presence of neither inhibitor during irradiation affected the initial degree of induction of γH2AX foci. However, the resolution of γH2AX foci to basal levels during recovery from IR occurred earlier and faster when PARG was inhibited and was delayed when PARP1/2/3 was inhibited. In control cells maximum levels of IR-induced DNA damage were reached at 10–30 min, resolution began after 30 min but was not significant until 6 h post-IR, and basal levels were restored within 24 h post-IR. In the presence of the PARG inhibitor, peak levels were reached at 10 min post-IR, resolution began between 10 and 30 min, was significantly different to control at 1 h post-IR, and basal levels were reached around 12 h after IR. In contrast, olaparib treated cells showed peak levels of DNA damage at 10 min post-IR, which after an initial small reduction remained relatively high for up to 6 h with significant resolution then occurring between 6 and 24 h after irradiation.

Together these data suggest that DNA double strand break repair is altered by olaparib or PDD00017273 however each agent may not function via the same mechanism.

### 3.3 PARG but not PARP1/2/3 inhibition promotes rapid activation of IR-induced DNA-PKcs, while both PARP and PARG delay resolution of IR-induced RAD51 foci

**Fig. 4:**
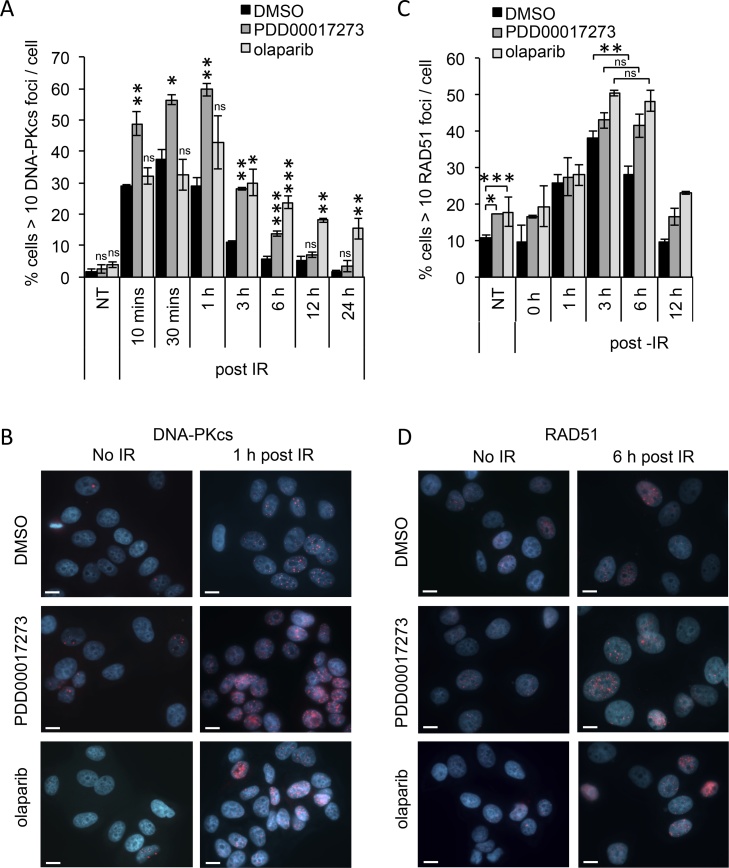
**PARG and PARP inhibitors have different effects on activation of DNA damage repair pathways following ionising radiation.** Percentage of cells displaying (**A**) >10 DNA-PKcs foci/cell and (**C**) >10 RAD51 foci/cell, in untreated (DMSO), PARG inhibited (0.3 μM PDD00017273), or PARP inhibited (1 μM olaparib) MCF-7 cells in the absence and at various times post 3 Gy ionising radiation (IR). Mean and SEM of three independent repeats is shown. Statistical significance calculated by two-sided Student’s *T*-test, where *, ** and *** represent p < 0.05, <0.01 and <0.001 respectively In (**A**) significance is compared with DMSO control at the equivalent time point and in (**C**) significance calculated compared with the sample indicated. (**B**) Example images of DNA-PKcs foci and (**D**) RAD51 foci (Red) each costained with DAPI. In all cases MCF-7 cells were incubated for 16 h with inhibitor before exposure to IR.

Radiation induces both single and double strand breaks in DNA however the kinetics of repair seen above suggest that it is changes to the repair of DNA DSBs that accounts for much of the difference between control and PDD00017273/olaparib treated cells. IR-induced DNA DSBs are repaired by two separate but complementary pathways – non-homologous end-joining (NHEJ) and homologous recombination repair (HRR). It is generally considered that repair by NHEJ is rapid, error prone and can occur at all stages of the cell cycle whereas HRR is slower, error free and is restricted to S- and G2-phases of the cell cycle [71]. We examined the relative contribution of each of these pathways to the repair of IR-induced DNA damage in the presence/absence of olaparib or PDD00017273, using activated DNA-dependent protein kinase, catalytic subunit (DNA-PKcs, pS2051) foci as a marker of NHEJ and RAD51 recombinase (RAD51) foci as a marker of HRR (Fig. 4). In control cells, DNA-PKcs foci rapidly increased in response to IR, peaked at 30 min and quickly resolved such that only 20% of the foci remained at 3 h post-IR. The remaining foci were then slowly resolved to basal levels by 24 h post-IR (Fig. 4A and B). In the same control cells, RAD51 foci levels increased slowly to peak at 3 h then decreased to basal levels by 12 h post-irradiation (Fig. 4C and D). In the presence of olaparib or PDD00017273, the appearance of IR-induced RAD51 foci was delayed compared with control cells but peaked at a similar 3 h post-IR. In inhibited cells, IR-induced RAD51 foci then persisted at peak levels until 6 h (no significant decrease in foci between 3 and 6 h in inhibited cells compared with a 25% decrease in control cells; p < 0.01), after which they were resolved and basal levels were restored by 12 h post-IR (Fig. 4C). In contrast to RAD51 foci, DNA-PKcs foci responded differently depending on whether PARP1/2/3 or PARG was inhibited. In the presence of olaparib, DNA-PKcs foci increased and peaked at similar levels, and with similar kinetics, to control cells (approximately 30% cells with greater than 10 foci/cell by 10 min post-IR). However, these foci then persisted at peak levels until 3–6 h post-IR (30% and 24% in PARP1/2/3 inhibited cells compared with 11% and 6% in control at 3 and 6 h respectively; p < 0.05 and p < 0.001 at each time point) with a significant percentage of cells containing high levels of foci even 24 h post-IR (Fig. 4A). On the other hand, in PARG inhibited cells there was a rapid induction of DNA-PKcs foci to almost double the peak level seen in control cells (49% compared with 29% respectively; p < 0.01). This increase persisted until 1 h post-IR, after 3 h approximately half had been resolved and then similar to controls, basal levels were restored by 12–24 h post-IR (Fig. 4A). Together these data suggest that although olaparib and PDD00017273 sensitize to IR, there may be a difference in the way cells respond to IR-induced DNA double strand breaks, with increased NHEJ activity being prompted in the presence of PARG inhibitor, but not following PARP1/2/3 inhibition. It is likely that it is the differential kinetics of activation of each pathway by olaparib or PDD00017273 following IR that contributes to the difference in resolution of γH2AX foci seen above. Interestingly, in the absence of IR, endogenous levels of RAD51 but not DNA-PKcs foci were increased by both inhibitors, suggesting that this PARG inhibitor induced NHEJ activity is specific to IR-induced DNA damage.

### 3.4 PARP and PARG inhibitors specifically increase IR-induced γH2AX positive micronuclei formation

**Fig. 5:**
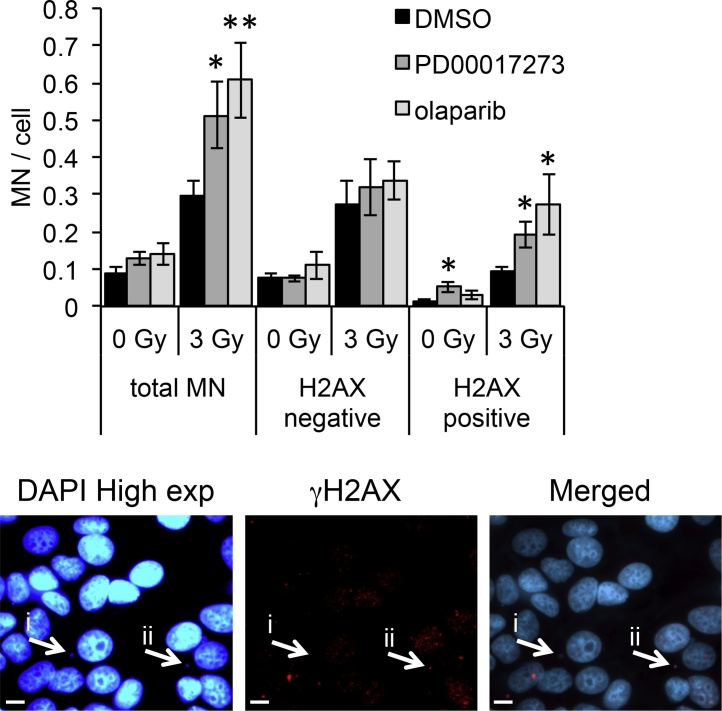
PARP and PARG inhibitors increase γH2AX positive micronuclei after ionising radiation. Micronuclei (MN) frequency in untreated (DMSO), PARG inhibited (0.3 μM PDD00017273), or PARP inhibited (1 μM olaparib) MCF-7 cells in the absence or 12 h post 3 Gy ionising radiation (IR). Mean and SEM of three independent repeats is shown. Statistical significance calculated by two-sided Student’s *T*-test compared with DMSO control under equivalent conditions, where * represents p < 0.05. Representative images depicting (i) γH2AX negative and (ii) γH2AX positive MN are shown below. A full data set of MN under each condition is shown in Supplementary Fig. 3.

The formation of micronuclei (MN) can occur due to errors in chromosome segregation during anaphase. IR induces γH2AX positive (+ve) micronuclei in cancer cells, perhaps as a result of unrepaired DSBs and broken chromosome ends being incorporated into IR-induced MN [72], or reflecting altered chromatin structures caused after illegitimate DNA repair [73]. We examined MN formation following IR in the presence or absence of olaparib or PDD00017273 (Fig. 5 and Supplementary Fig. 3). As expected radiation induced total MN formation significantly compared with untreated controls, with an average of 0.3 MN/cell compared with 0.1 MN/cell in DMSO treated samples (p < 0.001 comparing IR alone to no IR). This increase was greater in cells where PARP1/2/3 or PARG were inhibited with IR-induced MN levels of 0.5 and 0.6 MN/cell for PDD00017273 and olaparib respectively (p < 0.05 for each compared with IR alone). When γH2AX positive (+ve) and negative (−ve) MN were analysed the majority of IR-induced MN were −ve. Interestingly though it was the γH2AX +ve rather than −ve MN that were increased by PARP1/2/3 or PARG inhibition. This supports the idea that it is altered DNA repair that is responsible for radiosensitization by olaparib and PDD00017273.

### 3.5 PARG inhibition has a greater effect on IR-induced metaphase aberrations than inhibition of PARP1/2/3

**Fig. 6:**
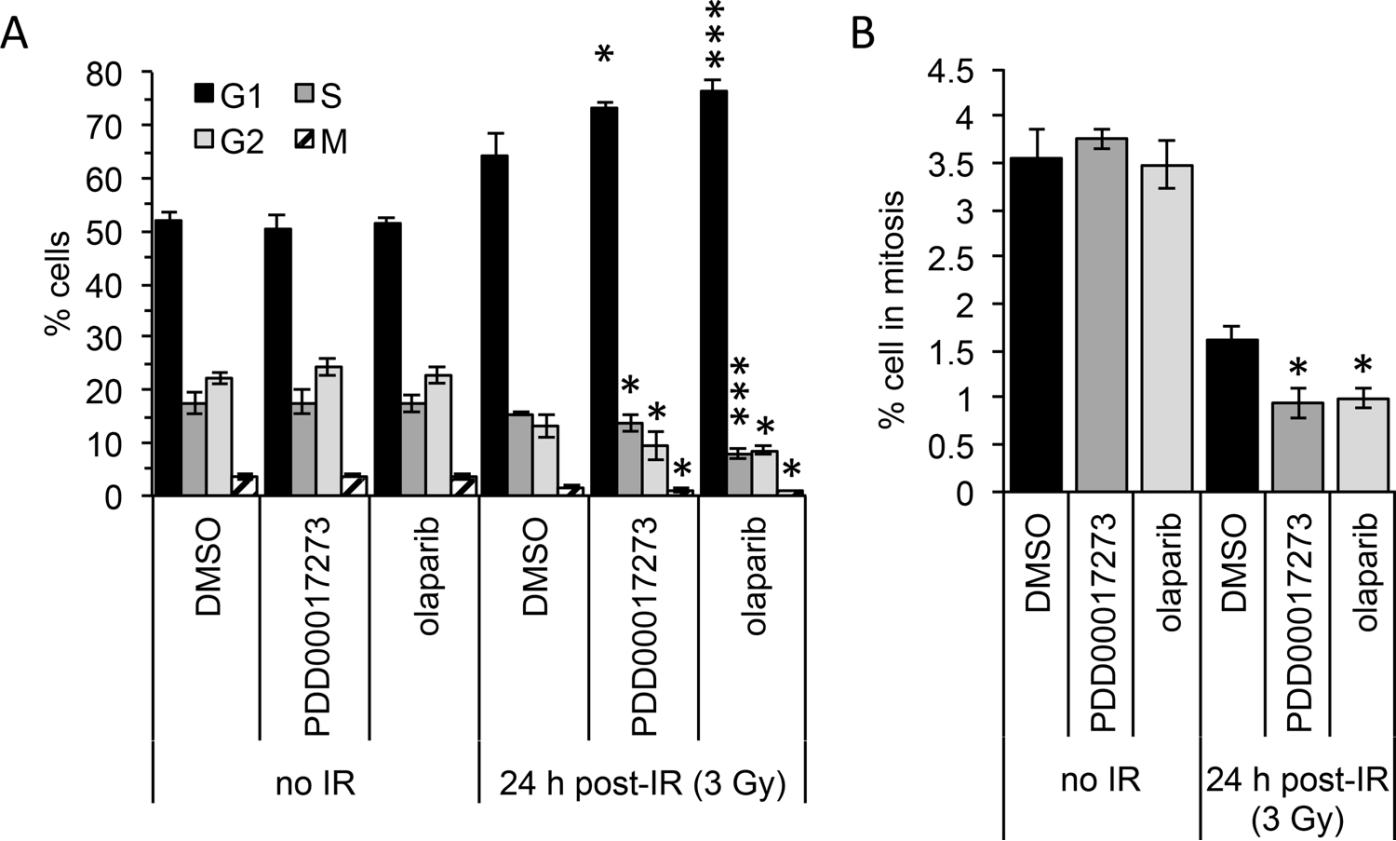
PARP and PARG inhibitors increase G1 arrest and reduce mitotic index following ionising radiation. Percentage of cells in each phase of the cell cycle as determined by FACS analysis of PI and pH3 stained MCF-7 cells untreated (DMSO), PARG inhibited (0.3 μM PDD00017273), or PARP inhibited (1 μM olaparib) in the absence or 24 h post 3 Gy ionising radiation (IR). For clarity (**A**) depicts all phases of cell cycle and (**B**) represents only the mitotic fraction (pH3 positive) from the same data set plotted on a different scale. Mean and SEM of three independent repeats is shown. Statistical significance calculated by two-sided Student’s *T*-test compared with DMSO control under equivalent conditions, where * and *** represent p < 0.05 and <0.001 respectively.

Previous reports of the effect of PARP1/2/3 inhibition on IR-induced cell cycle distribution are limited however radiosensitization is generally thought to be replication dependent [24], [27]. In addition there are conflicting reports regarding the effects of PARG depletion on IR-induced cell cycle checkpoints. For example, enhanced G2/M checkpoint arrest is seen in PARG depleted HeLa cells [51], while abrogation of this arrest is reported in lung cancer cells [64]. Here, the cell cycle profile of MCF-7 cells following IR in the presence or absence of olaparib or PDD00017273 was examined by flow cytometry. By co-staining for phosphorylation of H3 Serine 10 (pH3) the percentage of cells in mitosis could also be observed (Fig. 6). At the relatively low doses of radiation used here, in MCF-7 cells the predominant activated cell cycle checkpoint was the G1/S checkpoint [74]. Addition of the PARG inhibitor PDD00017273 enhanced the G1/S checkpoint arrest while reducing the number of cells in S, G2 and M phases of the cell cycle (p < 0.05, compared with equivalent phase in IR-treated non-inhibited cells). Olaparib had a similar effect but with a more predominant effect on S-phase (p < 0.001, compared with IR-treated non-inhibited cells). Neither PDD00017273 nor olaparib altered the cell cycle profile in the absence of IR.

**Fig. 7:**
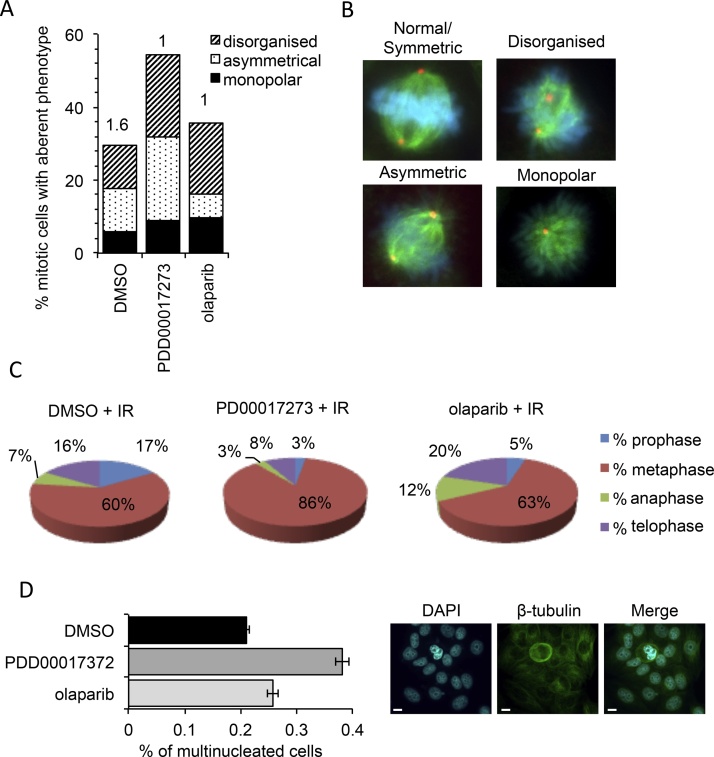
PARG inhibited mitotic cells accumulate in metaphase and feature increased aberrance. (**A**) Percentage of mitotic MCF-7 cells untreated (DMSO), PARG inhibited (0.3 μM PDD00017273), or PARP inhibited (1 μM olaparib) 24 h post 3 Gy ionising radiation (IR). Abnormal spindle defects are detected by immunofluorescent staining for β-tubulin (Green), pericentrin (Red) and DAPI (Blue), the value above the bars indicates the percentage of the total cell population in mitosis. (**B**) Representative images of aberrant phenotypes seen. (**C**) Distribution of cells in each phase of mitosis assessed from cells stained as above. (**D**) Percentage of multinucleated cells as stained above, example images shown to right.

Following IR both PARP and PARG inhibitors reduced the percentage of cells in mitosis (Fig. 6B, p < 0.05, compared with IR-treated non-inhibited cells). The fidelity of mitosis was tested by immunodetection of microtubules and centrosomes. Following IR in the presence of PARG inhibition, an increase in the percentage of mitotic cells with aberrant spindle formation was observed (Fig. 7A) including monopolar, asymmetric and disorgansied spindle formations (Fig. 7B). This was not seen following inhibition of PARP1/2/3 with olaparib. Aberrant mitosis was accompanied by an increase in the proportion of mitotic cells in prometaphase/metaphase (Fig. 7C). This suggests that despite the decrease in total cells in mitosis, PARG inhibition does effect mitotic progression in IR treated MCF-7 cells. The large shift in the proportion of cells in metaphase was not seen following IR with PARP inhibition, where similar to control, 63% of mitotic cells were in metaphase. In the absence of IR, PDD00017273 alone did not alter the percentage of cells in mitosis (Fig. 6B), nor did it effect progression through mitosis (Supplementary Fig. 4), suggesting at the doses used here it does not act directly as a spindle poison.

Tankyrases (PARP5a and PARP5b) function during mitosis [75], [76], [77], [78], [79], [80], [81]. Their action is likely to be reversed by PARG but it is not inhibited by olaparib. The incidence of aberrant mitosis and mitotic progression were therefore examined after incubation with a tankyrase inhibitor [68]. Following IR, the tankyrase inhibitor did phenocopy PDD00017273, in that it increased the incidence of aberrant spindles, however it did not lead to accumulation of cells in metaphase (Supplementary Fig. 5). Interestingly inhibition of tankyrases also sensitized to IR (Supplementary Fig. 6). While this was not to the extent seen with PD00017273, it does suggest that the likely mitotic function of PARG in the reversal of tankyrase activity may have a role to play in radiosensitization.

Concurrent with scoring aberrant mitosis, the percentage of cells with multinucleation was assessed. PARG inhibition also increased 2 fold the amount of IR induced multinucleation (Fig. 7E). Approximately 1000 cells were counted of which no cells were found to be multinucleate in the absence of IR regardless of PARP or PARG inhibition (data not shown).

## 4 Discussion

This is the first report of radiosensitization by a first in class, cell permeable, specific inhibitor of PARG, PDD00017273, and supports the proposal made by genetic studies [51], [62], [63], [64] that PARG inhibitors could be used clinically. In addition, we present the first direct comparison of the radiosensitizing effects of the PARP1/2/3 inhibitor, olaparib, with a PARG inhibitor. Sensitization to IR was of the same magnitude and, as expected, both functioned by altering the DNA damage response. However, the way in which each sensitized appeared to differ.

PARP1 and PARP2 function at collapsed replication forks [18], [82] and radiosensitization by inhibitors of PARPs is thought to be replication dependent [24], [27], with an increase in γH2AX and RAD51 foci reported. Similar to these reports, here IR-induced γH2AX persisted at later times post-IR in olaparib treated compared with control cells and a greater number of RAD51 foci were seen. In addition, there were more DNA-PKcs foci at later times post-IR in olaparib treated than in control cells. These data are consistent with the idea that replication associated DNA breaks are repaired at later times [18] and/or that the presence or absence of PARPs 1/2 and 3 can alter the recruitment of the non-homologous end-joining factors XRCC6/XRCC7 (KU70/80) to DSBs [15], [83].

In contrast, inhibition of PARG resulted in faster repair of IR-induced DNA damage and concomitant rapid activation of significantly higher levels of DNA-PKcs. NHEJ involving DNA-PKcs is thought to predominate during G1 phase of the cell cycle, while during S and G2 both NHEJ and HR can function [84], [85]. Our data suggest that prolonged activation of PAR can increase recruitment of NHEJ to DNA damage during G1, and/or alter the balance of NHEJ/HR to DNA damage in other phases of the cell cycle. At later time points PARG inhibitors also led to higher levels of RAD51 foci perhaps indicative of a separate role for PARG at collapsed replication forks [53], [66]. This functional difference at classical DSBs and replication fork associated DSBs is supported by that fact that in the absence of any exogenous DNA damage, PARG inhibitors were seen to activate HR but not NHEJ.

There are conflicting reports of the effect of PARG depletion on cell cycle progression, with one report of IR treated HeLa cells having increased G2/M arrest and accumulation of cells in metaphase [51], while another in lung (A427) and prostate (PC-14) cancer cell lines demonstrated suppression of the G2/M checkpoint [64]. Clearly the mutational landscape of individual cell lines will affect their response to a PARG inhibitor and/or IR. However, here in MCF-7 breast cancer cells, while PARG inhibition resulted in a reduced G2/M population and a reduced mitotic index, those cells in mitosis had an increased incidence of aberrant mitotic figures and a higher proportion of mitotic cells were in metaphase than in control cells. Interestingly, PARP inhibition also reduced the IR-induced G2/M population, however no increase in aberrant mitotic figures or metaphase was seen, indicating that the aberrant mitotic phenotypes were PARG specific. The PARP inhibitor olaparib is considered selective for PARP1/2/3 [67], while PARG is predicted to reverse the activity of a range of poly(ADP-ribosyl)ating enzymes including tankyrases (TNKS/PARP5a and TNKS2/PARP5b). Tankyrases are required for spindle integrity during mitosis through PARylation of nuclear mitotic apparatus protein 1 (NUMA1). Thus failure to cleave the PAR from NUMA1 (installed by the tankyrases) may give the observed results [86]. Here inhibition of tankyrases resulted in an increase in IR-induced aberrant mitotic phenotypes and led to a small increase in radiosensitivity, thus, it is possible that the PARG inhibitor specific mitotic phenotypes observed are due to interruption of tankyrase function. Alternatively, given the role of PARP3 during mitosis [86], it is possible that preventing addition of PAR by PARP3 has functional effects that inhibition of PARP3 does not. Finally, aberrance during mitosis could be the result of increased and inappropriate NHEJ.

Using an early moderate pan-PARP inhibitor (5-hydroxyisoquinolin-1-one) and perhaps more relevantly an exogenously expressed transdominant PARP-1 DNA binding domain, PARP-1 was demonstrated to positively regulate p53 transactivation function in response to IR and therefore allow MCF-7 cells to overcome the p53 dependent G1 checkpoint [87]. Here, although a predominant IR-induced G1 arrest was seen in control cells neither olaparib nor PDD00017273 could overcome this arrest, rather it was augmented. It is possible that differences in the scheduling of inhibition and IR account for this, such that the increased pre-incubation time carried out here, allows p53 to overcome PARP/PARG inhibition. Future studies of p53 function in moderating radiation response upon the PARG/PARG inhibition will be important for future clinical application. In MCF-7 cells the number of cells in G1 is further increased as cells are released from the transient G2/M arrest and pass into G1 [88]. It is tempting to speculate that in cells where the DNA damage repair pathways are altered (i.e. following PARP or PARG inhibition), cells with damaged DNA can persist into G1 where they die of mitotic catastrophe. Supportive of this hypothesis we see increased IR-induced γH2AX positive micronuclei and increased numbers of multinucleated cells.

## 5 Conclusion

In summary, previously we demonstrated the use of the PARG inhibitor PDD00017273 for specific killing of cells defective in certain HR proteins including BRCA1/2 [66]. Here, the same inhibitor is shown to radiosensitize. This is the first report of radiosensitization by a PARG inhibitor and adds to the growing evidence that like PARP, inhibition of PARG has clinical potential. However, when looking at the mechanism by which sensitization occurs there are clear differences between PARP and PARG inhibition, and it is important that further investigation into these differences is undertaken.

## Author contributions

HB conceived, designed and initiated the study. PG, JN, EG and CD performed the experiments. AN designed and synthesised the tankyrase inhibitor. DJ and KS designed and synthesised PDD00017273. PG and HB wrote the manuscript. All authors discussed the results and commented on the manuscript.

## Funding

This work was supported by Breast Cancer Now [grant number PR016], and CRUK grant [number C5759/A17098]. Microscopy was performed on equipment purchased by Welcome Trust grant WT093134AIA and MRC SHIMA award MR/K015753/1.

## Conflict of interest

The authors declare no competing financial interest.
