# Supplementary material for: Radiosensitization with an inhibitor of poly(ADP-ribose) glycohydrolase: A comparison with the PARP1/2/3 inhibitor olaparib
Source: DNA Repair (Amst). 2018 Jan;61:25–36. doi: 10.1016/j.dnarep.2017.11.004 (PMC5765821; doi:10.1016/j.dnarep.2017.11.004)
Supplement: Supplementary file 1 [file mmc1.pdf]

Supplementary Figure S1

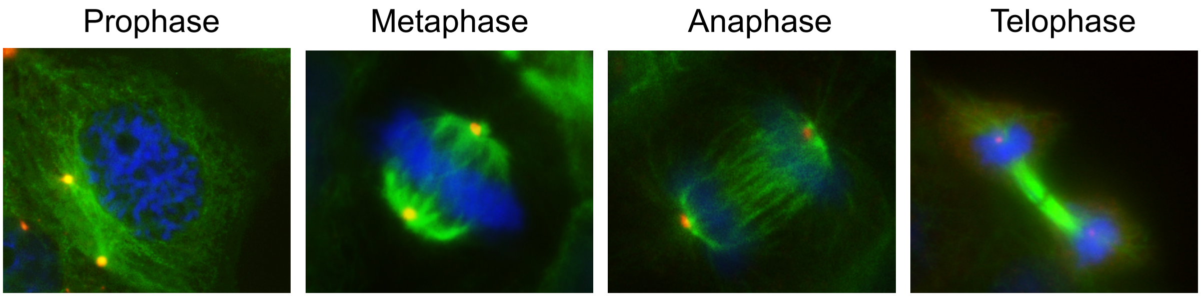

Supplementary Figure S1. Example images of each stage of mitosis.
Immunofluorescent staining of MCF-7 cells for β-tubulin (Green), pericentrin (Red) and DAPI (Blue).

Supplementary Figure S2

MDA-MB-231

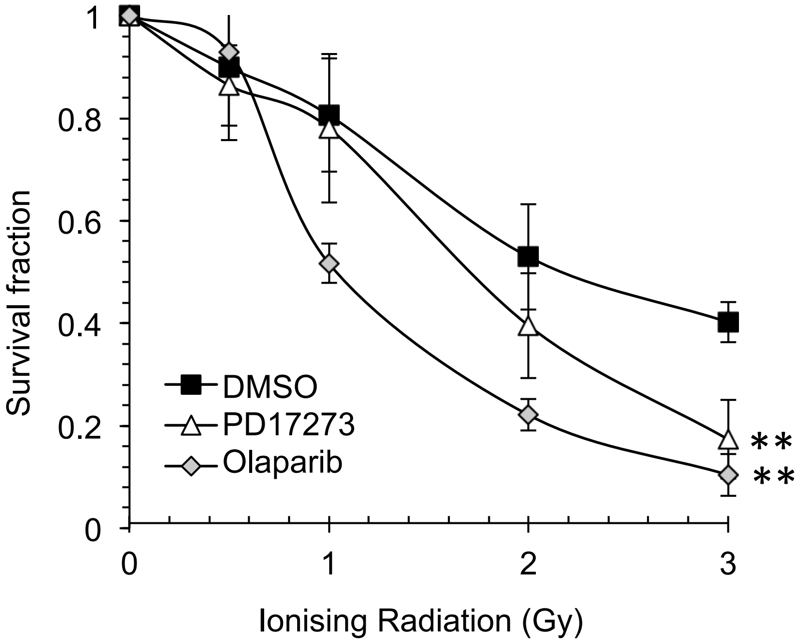

Supplementary Figure S2. Inhibition or depletion of PARP or PARG increases sensitivity to ionising radiation. Survival fraction of MDA-MB-231 cells untreated (DMSO), treated with PARG inhibitor (PDD00017273), or PARP inhibitor (olaparib). Survival was measured by clonogenic survival assay. Mean and standard deviation of three independent repeats are shown. Statistical significance calculated by Student's T-test, c.f to DMSO where ** represents $p<0.01$.

Supplementary Figure S3

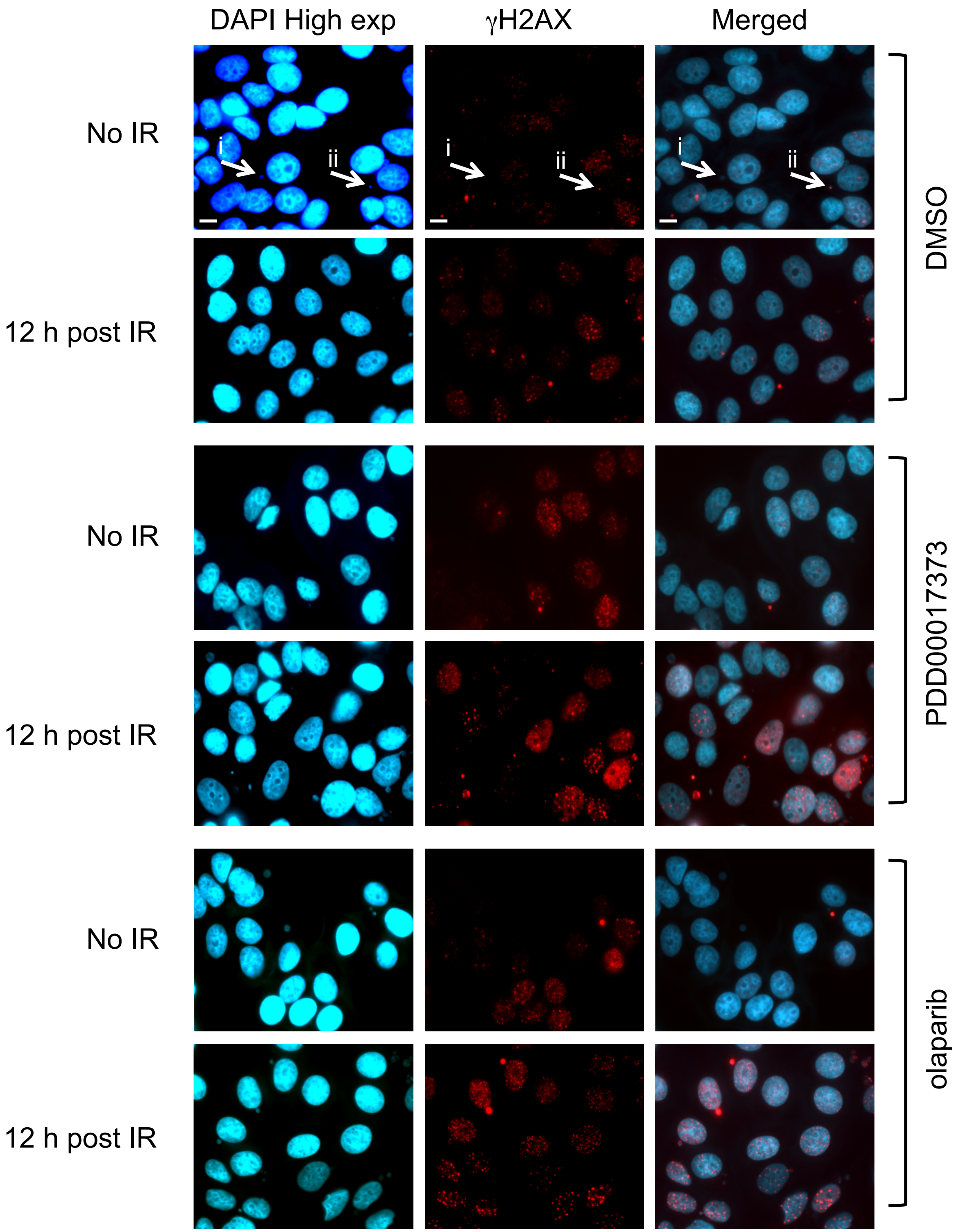

Supplementary Figure S3. PARP and PARG inhibitors increase γH2AX positive micronuclei after ionising radiation. Micronuclei (MN) frequency in untreated (DMSO), PARG inhibited (0.3 μM PDD00017273), or PARP inhibited (1 μM olaparib) MCF-7 cells in the absence or 12 h post 3 Gy ionising radiation (IR). Representative images depicting (i) γH2AX negative and (ii) γH2AX positive MN are shown. The top panel is repeated from main text for clarity.

Supplementary Figure S4

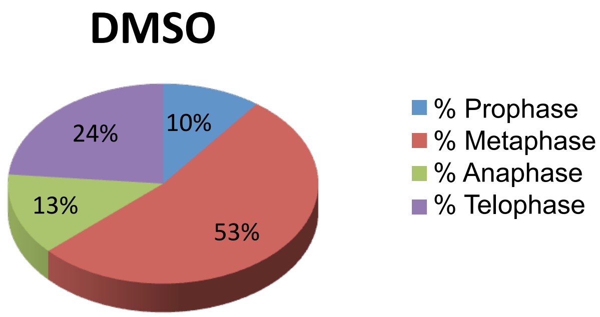

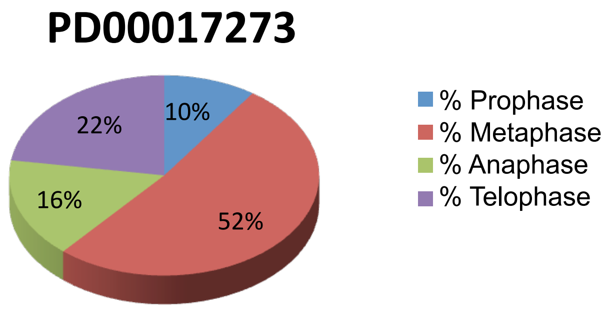

Supplementary Figure S4. PARG inhibition alone did not alter mitotic progression. Distribution of MCF-7 cells in each phase of mitosis 24 h post ionising radiation as detected using immunofluorescent staining for β-tubulin (Green), pericentrin (Red) and DAPI (Blue). Control (DMSO) and treated with PDD00017273 (PD).

Supplementary Figure S5

A

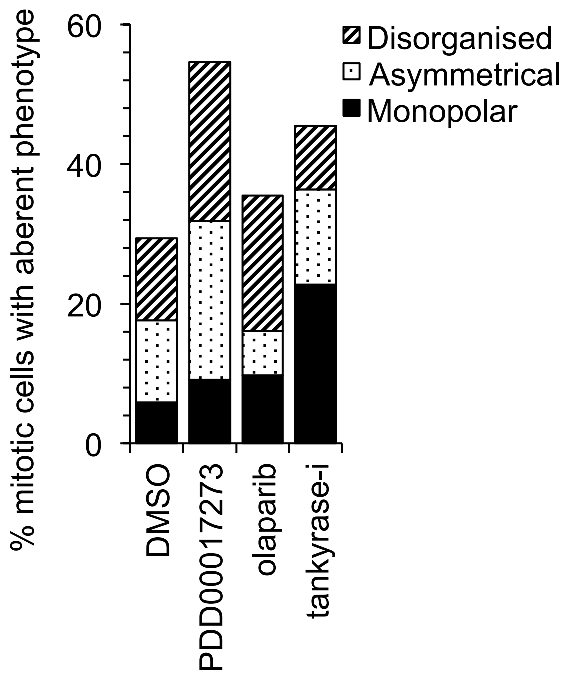

B

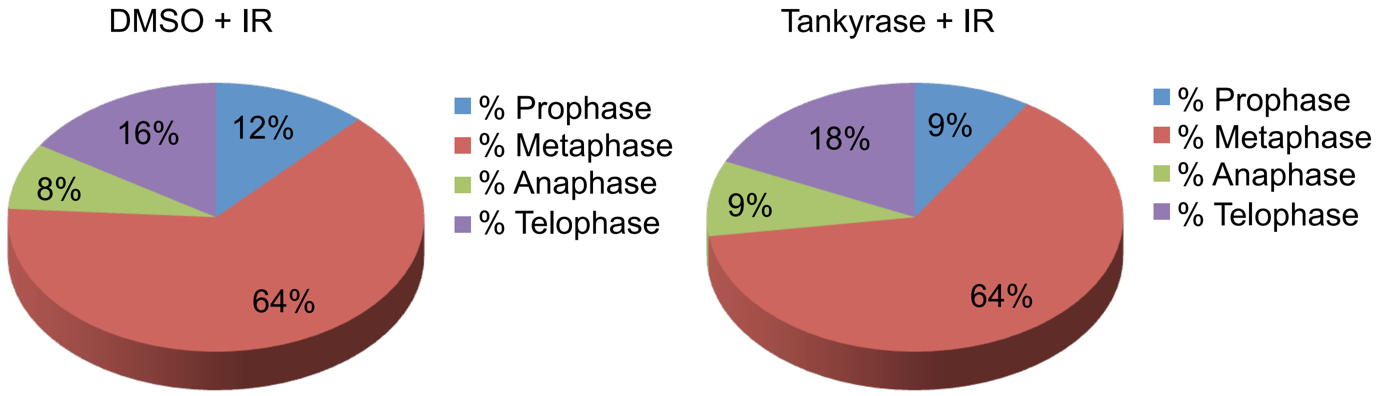

Supplementary Figure S5. Tankyrase-inhibited mitotic cells feature increased aberrance but do not accumulate in metaphase. (**A**) Percentage of mitotic MCF-7 cells treated with tankyrase inhibitor 24 hours post 3 Gy ionising radiation (IR) showing abnormal spindle defects as detected using immunofluorescent staining for β-tubulin (Green), pericentrin (Red) and DAPI (Blue). (**B**) Distribution of cells in each phase of mitosis assessed from cells stained as above.

Supplementary Figure S6

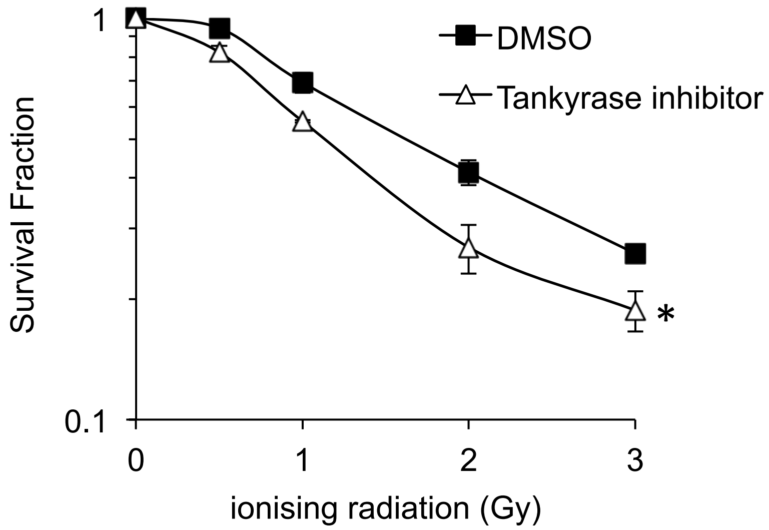

Supplementary Figure S6. Inhibition of tankyrases increases sensitivity to ionising radiation. Survival fraction of MCF-7 cells untreated (DMSO), treated with 5 μM Tankyrase inhibitor. Survival was measured by clonogenic survival assay. Mean and standard deviation of two independent repeats are shown. Statistical significance calculated by Student's T-test, c.f to DMSO where * represents $p<0.05$.
